# Fatty Acid Metabolite Profiling Reveals Oxylipins as Markers of Brown but Not Brite Adipose Tissue

**DOI:** 10.3389/fendo.2020.00073

**Published:** 2020-02-21

**Authors:** Sebastian Dieckmann, Stefanie Maurer, Tobias Fromme, Cécilia Colson, Kirsi A. Virtanen, Ez-Zoubir Amri, Martin Klingenspor

**Affiliations:** ^1^Chair for Molecular Nutritional Medicine, TUM School of Life Sciences, Technical University of Munich, Freising, Germany; ^2^EKFZ - Else Kröner-Fresenius Center for Nutritional Medicine, Technical University of Munich, Freising, Germany; ^3^ZIEL Institute for Food and Health, TUM School of Life Sciences, Technical University of Munich, Freising, Germany; ^4^Université Côte d'Azur, CNRS, Inserm, iBV, Nice, France; ^5^Turku PET Centre, Turku University Hospital, University of Turku, Turku, Finland

**Keywords:** adipose tissue, browning, thermogenesis, PUFA (polyunsaturated fatty acid), n-6 fatty acid, n-3 fatty acid, oxylipin

## Abstract

Metabolites of omega-6 and omega-3 polyunsaturated fatty acids are important signaling molecules implicated in the control of adipogenesis and energy balance regulation. Some of these metabolites belonging to the group of oxylipins have been associated with non-shivering thermogenesis in mice mediated by brown or brite adipose tissue. We aimed to identify novel molecules with thermogenic potential and to clarify the relevance of these findings in a translational context. Therefore, we characterized and compared the oxylipin profiles of murine and human adipose tissues with different abundance of brown or brite adipocytes. A broad panel of 36 fatty acid metabolites was quantified in brown and white adipose tissues of C57BL/6J mice acclimatized to different ambient temperatures and in biopsies of human supraclavicular brown and white adipose tissue. The oxylipin profile of murine brite adipose tissue was not distinguishable from white adipose tissue, suggesting that adipose tissue browning *in vivo* is not associated with major changes in the oxylipin metabolism. Human brown and white adipose tissue also exhibited similar metabolite profiles. This is in line with previous studies proposing human brown adipose tissue to resemble the nature of murine brite adipose tissue representing a heterogeneous mixture of brite and white adipocytes. Although the global oxylipin profile served as a marker for the abundance of thermogenic adipocytes in bona fide brown but not white adipose tissue, we identified 5-HETE and 5,6-EET as individual compounds consistently associated with the abundance of brown or brite adipocytes in human BAT and murine brite fat. Further studies need to establish whether these candidates are mere markers or functional effectors of thermogenic capacity.

## Introduction

Obesity is one of today's major health burdens with a steadily increasing prevalence. It is characterized by excessive fat accumulation and unhealthy expansion of white adipose tissue (WAT) associated with severe comorbidities such as type 2 diabetes and cardiovascular diseases. Obesity is the consequence of a chronic positive energy balance, a state where energy intake exceeds energy expenditure. A major obstacle of obesity management is the maintenance of a given body weight loss, since weight loss is accompanied by a notable and persistent decrease in energy expenditure (1, 2). This decrease in energy expenditure is hardly compensated by physical activity, the only available strategy to increase energy expenditure so far. Consequently, other means to increase energy expenditure are in demand. Thermogenic tissues such as brown (BAT) and brite adipose tissue are promising targets. Brite adipose tissue, in contrast to BAT, is an inducible type of fat originating from the recruitment of brown-like, so called brite (or beige) cells with thermogenic properties in WAT. Both tissues dissipate chemical energy from fatty acids and glucose to generate heat, thus increasing energy expenditure. This non-shivering thermogenesis is mediated by uncoupling protein 1 (UCP1). It is naturally activated upon cold exposure to defend body temperature and during eating to promote meal termination (3). Although the presence of functional BAT has been confirmed in adult humans (4–6), humans mostly live under thermoneutral conditions (7). Therefore, BAT activation in humans is mostly associated with food intake, whereas cold-induced activation is less prevalent. BAT volume and activity negatively correlate with BMI (8), suggesting a lower abundance of active BAT in overweight and obese compared to lean subjects. Consequently, the therapy of obesity by means of BAT and brite fat not only requires strategies to activate it but also to increase its abundance. Several natural compounds and drugs are associated with the activation and recruitment of BAT in mice and humans (9, 10). Among these are metabolites of omega-6 and omega-3 polyunsaturated fatty acids (PUFA). Oxygenated PUFA metabolites, belonging to the group of oxylipins, are important signaling molecules implicated in the control of adipogenesis and energy balance regulation (11). These potent and short-lived metabolites are generated by a series of enzymatic steps involving one of three enzyme classes—cyclooxygenase (COX), lipoxygenase (LOX) or cytochrome P450 (CYP) (12). Some oxylipins have been associated with the browning of adipose tissues. The COX derived ARA metabolites prostaglandin E2 (PGE2) and prostacyclin (PGI2) facilitate the formation of brite adipocytes *in vitro* (13–16). The oxylipin 12-hydroxyeicosapentaenoic acid (12-HEPE), identified in a PUFA metabolite screen in murine serum samples, facilitates glucose uptake into brown adipocytes (17). In a similar approach, increased levels of 12,13-dihydroxy-9Z-octadecenoic acid (12,13-diHOME) were identified in oxylipin profiles of human serum after cold acclimatization (18). This oxylipin increases fatty acid uptake into brown adipocytes and presumably UCP1 expression (18). Furthermore, a second class of PUFA derived metabolites, the endocannabinoids, are suggested to be involved in the negative regulation of BAT activity in mice (19). Thus, several lines of evidence suggest PUFA-derived metabolites to be involved in the recruitment and activity of thermogenic cells in mice and humans. The aim of the current study was to characterize the oxylipin profiles of murine and human adipose tissues with different abundance of brown and brite adipocytes to identify novel molecules with thermogenic potential in a translational context.

We quantified a panel of 36 fatty acid metabolites in brown and white adipose tissues of C57BL/6J mice acclimatized to different ambient temperatures and in biopsies of human supraclavicular brown and white adipose tissue. Our results reveal the global oxylipin profile of bona fide brown but not brite adipose tissue as a marker for the abundance of brown adipocytes. Moreover, we identified 5-HETE and 5,6-EET as individual compounds associated with the abundance of brown or brite adipocytes in both human BAT and murine brite fat.

## Materials and Methods

### Animal Experiments

Eight-week-old male C57BL/6J mice were housed in climate cabinets (HPP750 life, Memmert) at 23°C and 55% humidity with a 12/12 h light/dark cycle. Mice were provided *ad libitum* access to water and a control diet (Ssniff, Cat# S5745-E720). After an adaptation phase of 3 weeks, mice were assigned to one of two groups and transferred to preconditioned cabinets at 5 or 30°C. After 1 week, mice were killed by CO_2_ exposure and tissues were immediately dissected, snap frozen in liquid nitrogen, and stored at −80°C until further processing. The experiment was performed according to the German animal welfare law with permission from the district government of Upper Bavaria (Regierung von Oberbayern, reference number ROB-55.2-2532.Vet_02-16-166).

### Human Subjects

Paired biopsies of BAT and WAT were obtained from the supraclavicular region of 14 healthy male and female subjects. A detailed description of the biopsy procedure and of anthropometric characteristics of this study cohort has been published previously (20). Depending on the size of the specimens obtained, BAT and WAT were either entirely subjected to RNA isolation or grinded in liquid nitrogen to obtain aliquots used for both metabolite analysis and RNA isolation.

### Oxylipin and Endocannabinoid Profiling

Murine interscapular BAT, inguinal WAT and human supraclavicular fat biopsies were grinded in liquid nitrogen. Aliquots of 23–140 mg were subjected to oxylipin and endocannabinoid analysis, which was conducted at the Metatoul lipidomic platform (INSERM UMR1048, Toulouse, France), certified to ISO 9001:2015 standards. Metabolite abundance was normalized to tissue mass.

### RNA Isolation and Quantitative Real-Time PCR (qRT-PCR)

RNA isolation from murine inguinal WAT and supraclavicular BAT as well as human adipose tissues was performed with TRIsure™ (Bioline, Cat# BIO-38032) according to the manufacturer's instructions. Precipitated RNA was transferred to spin columns (SV Total RNA Isolation System, Promega, Cat# Z3105), centrifuged for 1 min with 12,000 × g and further processed according to the supplier's instructions. RNA concentration was determined spectrophotometrically (Infinite 200 PRO NanoQuant, Tecan). Generation of cDNA was performed with 1 μg RNA (SensiFAST™ cDNA Synthesis Kit, Bioline, Cat# BIO-65053). qRT-PCR was performed in a 384 well plate format with the LightCylcer 480 system (Roche Diagnostics) in a total reaction volume of 12.5 μl containing 6.25 μl 2x SensiMix SYBR no-ROX (Bioline, Cat# QT650-05), 250 nM forward and reverse primers and 1 μl template cDNA. Murine primers (Ucp1 5′-TCTCTGCCAGGACAGTACCC-3′ and 5′-AGAAGCCCAATGATGTTCAG-3′, Tf2b 5′-TGGAGATTTGTCCACCATGA-3′ and 5′-GAATTGCCAAACTCATCAAAACT-3′) and human primers (UCP1 5′-GGAGGCCTTTGTGAAAAACA-3′ and 5′-CTTGAAGAAAGCCGTTGGTC-3′, TF2B 5′-GCTGTGGAACTGGACTTGGT-3′ and 5′-AGTTTGTCCACTGGGGTGTC-3′) were produced by Eurofins MWG Operon. Expression of Ucp1 was normalized to transcription factor 2b (Tf2b) expression.

### Statistical Analysis

All statistical analyses were performed using R-Studio (version 1.2.5019) with R version 3.6.1. Principal component analysis was performed with the R packages factoextra (version 1.0.5) and FactoMineR (version 1.42). Other statistical tests were calculated with the R package ggpubr (version 0.2.3). Wilcoxon test was performed for all group comparisons, after checking the assumption of normal distribution with Shapiro–Wilk test. *P* < 0.05 were deemed statistically significant. The appropriate statistical test, paired, or unpaired is mentioned for each figure.

## Results

**Figure 1:**
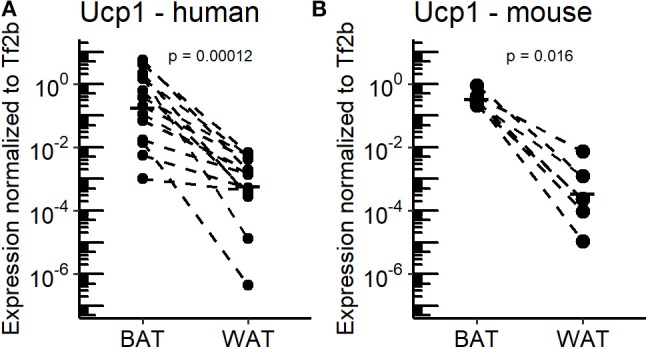
Ucp1 expression in BAT and WAT of the murine and human study cohorts. **(A)** Uncoupling protein 1 (Ucp1) mRNA expression in supraclavicular brown adipose tissue (BAT) and white adipose tissue (WAT) of human subjects (*n* = 14). **(B)** Ucp1 mRNA expression in inguinal WAT and supraclavicular BAT of mice housed at 30°C for 1 week (*n* = 7). *P*-values are derived from paired Wilcoxon test.

Adaptive, non-shivering thermogenesis is the key functional difference that discriminates mammalian BAT and WAT. Since almost a decade, the rediscovery of functional BAT in adult humans has intensified efforts to characterize the molecular properties of human adipose tissues and to identify novel thermogenic effectors intended for therapeutic use. Within this scope, oxylipins appear to be a promising class of endogenous compounds affecting the function and recruitment of thermogenic adipocytes in cultured cells of human and murine origin (11). In the course of this study, we further elucidated the association of these metabolites with the recruitment of thermogenic brown and brite adipocytes in a translational context. To this end, we subjected BAT and WAT of murine and human origin to metabolite profiling and analyzed the data in consideration of the tissues' thermogenic properties. Human BAT and WAT biopsies were obtained from the supraclavicular region subsequent to PET imaging under cold-exposed conditions (20). Humans live within thermoneutral conditions most of their life (7). Thus, for a more appropriate comparison between mice and humans we acclimatized C57BL/6J mice to 30°C for 1 week to mimic the thermal environment of humans. In order to confirm the thermogenic potential of BAT vs. WAT in both humans and mice, Ucp1 mRNA expression was quantified as a surrogate marker for the abundance of thermogenic competent adipocytes. As expected, all BAT specimens were characterized by considerably higher Ucp1 mRNA levels compared to WAT with a wide range of inter-individual variation (Figures 1A,B). However, mean Ucp1 mRNA expression in human and murine BAT was 544- and 255-fold higher compared to WAT, respectively. Consequently, human and murine BAT harbor more brown adipocytes than WAT.

### The Abundance of Adipose Tissue Oxylipins Differs Between Mice and Humans

**Figure 2:**
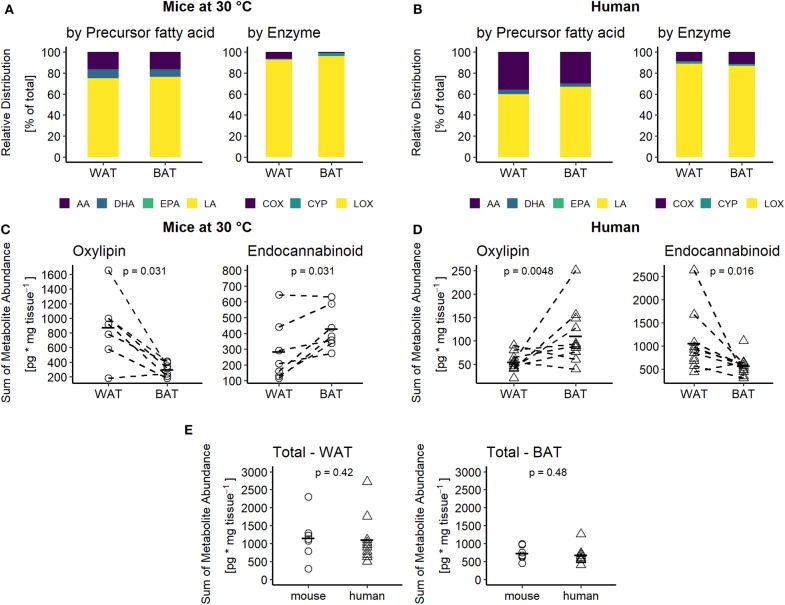
Adipose tissues of mice and men are comparable in terms of oxylipins composition but not abundance. Relative distribution of oxylipins categorized by their common fatty acid progenitor (left) or enzymatic synthesis pathway (right) for **(A)** mice at 30°C and **(B)** humans. Total sum of oxylipins (left) or endocannabinoids (right) for each individual **(C)** mouse (*n* = 7) or **(D)** human subject (*n* = 10 for WAT and *n* = 11 for BAT). **(E)** Total sum of combined oxylipins and endocannabinoids in murine and human WAT and BAT. Statistical analysis paired Wilcoxon test **(C)** and unpaired Wilcoxon test **(D,E)**.

To elucidate the regulation of oxylipin production in BAT and WAT, we quantified a broad panel of 33 metabolites representing major oxylipin classes produced by mammalian tissues. Additionally, we quantified the levels of 3 AA-derived endocannabinoids. The oxylipin panel encompasses COX, LOX and CYP-derived metabolites generated by the conversion of arachidonic acid (AA), its n-6 precursor linoleic acid (LA), and the n-3 fatty acids eicosapentaenoic acid (EPA) and docosahexaenoic acid (DHA). Within this setting, LA-derived metabolites were most abundant, while n-3 derived metabolites had a relatively low abundance in inguinal WAT and interscapular BAT of mice (Figure 2A). This high abundance of LA-derived metabolites was reflected in a high percentage of LOX-derived metabolites (Figure 2A). In human adipose tissues, the relative abundance followed a slightly different pattern. In both human BAT and WAT, AA-derived metabolites produced via the COX-pathway accounted for a higher percentage of total oxylipin abundance compared to murine fat, which proportionally reduced the relative levels of LA and DHA-derived metabolites (Figures 2A,B). The contribution of EPA-derived oxylipins to the oxylipins pool was negligible in adipose tissues of both species. Of note, the contribution of CYP derived oxylipins in mice was higher in interscapular BAT compared to inguinal WAT while in humans no notable difference was observed. Despite this similar composition of the oxylipin pools in WAT and BAT, the total abundance of all oxylipins in BAT vs. WAT in mice was significantly lower while it was significantly higher in human BAT vs. WAT (Figures 2C,D). These differences are primarily attributed to changes in the abundance of the LA-derived oxylipins 9- and 13-HODE. These two oxylipins are the predominant species in WAT and BAT, accounting for at least 60% of the total oxylipin pool in adipose tissues of both species (Figures 2A,B). Endocannabinoids represent another class of fatty acid metabolites with potential effects on Ucp1 dependent thermogenesis (19). Interestingly, the total abundance of the three endocannabinoids was lower in murine WAT vs. BAT but higher in human WAT vs. BAT, while we observed the exact opposite for total oxylipin abundance (Figures 2C,D). Ultimately, considering the combined pool of oxylipins and endocannabinoids there was no difference in total metabolite abundance between murine and human WAT or BAT (Figure 2E). Conclusively, mice and humans are similar in terms of the total production of PUFA metabolites. However, these tissues seem to differ in the partitioning of PUFA metabolism.

### The Global Oxylipin Profile Is a Surrogate Marker for the Abundance of Brown but Not Brite Adipocytes

**Figure 3:**
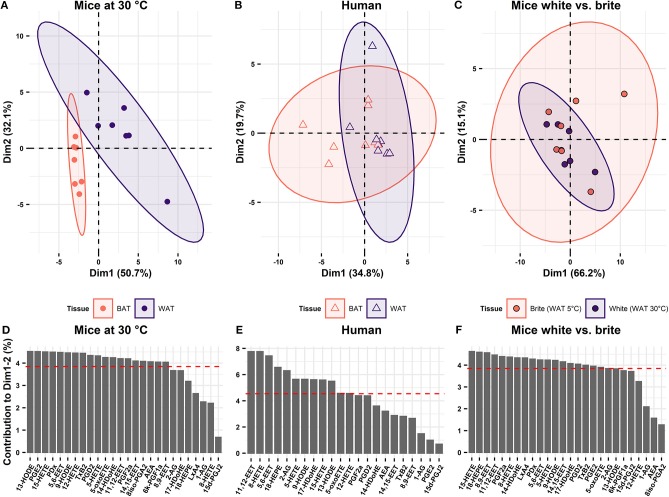
Oxylipin profiles distinguish BAT but not brite adipose tissue from WAT. Principal component analysis of the oxylipins in BAT and WAT showing the two first principal components (Dim1 and Dim2) in **(A)** mice at 30°C (*n* = 7) and **(B)** human (*n* = 8). **(C)** Principal component analysis of oxylipins in murine WAT acclimatized to 5 or 30°C (*n* = 7). Contribution of the single variables to Dim1 and Dim2 for **(D)** mice at mice at 30°C, **(E)** human, and **(F)** murine WAT acclimatized to 5 or 30°C for 1 week. Red dashed line indicates the average contribution of all variables.

As oxylipin abundance differs between WAT and BAT in both mice and humans, we investigated, whether oxylipins may serve as discriminative markers for the two tissues. Therefore, we applied principal component analysis (PCA) on the metabolite data of BAT and WAT. In mice, principal components 1 and 2 together explained 82.8% of the variability between WAT and BAT. In a continuum of these principal components, murine WAT and BAT formed distinct and separate clusters (Figure 3A). Thus, murine BAT and WAT can be distinguished by their characteristic oxylipin patterns. In humans, principal components 1 and 2 explained considerably less of the variation between BAT and WAT (54.5%). A distinction of human BAT and WAT according to their specific oxylipin patterns was not possible (Figure 3B). This was reflected in the analysis of the combined human and murine data set. In this analysis the two species formed distinct clusters separating the murine tissues while human BAT and WAT could not be distinguished (Supplementary Figure 1A). In contrast to murine BAT, human BAT constitutes a complex, interwoven mixture of both brown and white adipocytes. Consequently, oxylipin patterns established from human tissues may not represent differences on the cellular level of individual brown and white adipocytes and lack discriminative power (Figure 3B). To overcome this limitation, we transferred oxylipin patterns established from murine BAT and WAT and plotted human oxylipin levels according to these murine principal components. However, human BAT and WAT remained indistinguishable (Supplementary Figure 1B). Surprisingly, the reverse strategy, i.e., plotting murine oxylipin levels according to principal components of human oxylipin variation, murine BAT and WAT could be well-separated (Supplementary Figure 1C). This indicates that oxylipins in humans do not *per se* lack the variability observed in murine tissues but fail to sharply distribute into the categories BAT and WAT. The lack of discrimination of human supraclavicular BAT and WAT oxylipin patterns are in line with the observation that human supraclavicular BAT does not resemble the characteristics of classical BAT in conventional laboratory mice but rather displays a brite phenotype (7, 21). Indeed, brite adipose tissue obtained from mice housed at 5°C for 1 week and human supraclavicular BAT were both characterized by increased UCP1 expression compared to WAT (Supplementary Figure 2 and Figure 1A). Murine brite adipose tissue contained a mixed population of unilocular white and multilocular brown/brite cells (Supplementary Figure 3), similarly to the phenotype reported from human supraclavicular BAT (7). We investigated this by comparing oxylipin profiles of inguinal WAT of mice acclimatized to either 30°C (white adipose tissue) or 5°C (brite adipose tissue). In this comparison, the principal components 1 and 2 explained a large proportion (81.3%) of the variation between brite and white adipose tissue (Figure 3C). However, murine brite and white adipose tissue could not be separated from one another by oxylipin patterns (Figure 3C), although both formed distinct populations separate from BAT (Supplementary Figure 1D). Interestingly, BAT of mice acclimatized to 5 or 30°C also formed distinguishable populations (Supplementary Figure 1D). This suggests the global BAT oxylipin profile as surrogate marker of the abundance of brown adipocytes, since BAT of 5°C acclimatized mice contained more multilocular brown adipocytes than BAT of mice housed at 30°C. Conclusively, the oxylipin profiles of adipose tissues allow the discrimination of bona fide BAT and WAT composed of homogenous populations of brown and white adipocytes, respectively. However, it is either unsuitable to distinguish tissues harboring both types of cells or unable to distinguish brite from white adipocytes. Thus, the oxylipin profile can serve as a surrogate marker for brown adipocyte abundance in murine BAT but not murine brite fat or human BAT.

### The Oxylipins 5-HETE and 5,6-EET Are Potential Markers of Brown Adipocyte Abundance in BAT in Mice and Humans

**Figure 4:**
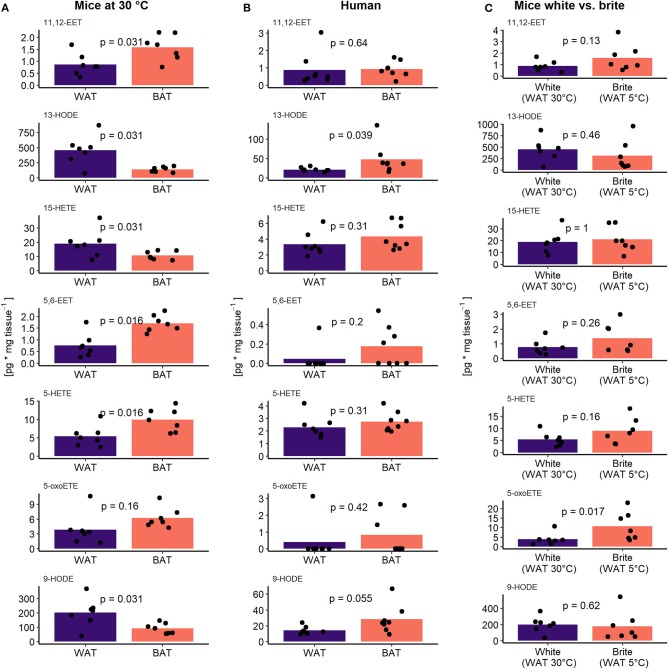
5-HETE and 5,6-EET are regulated similar between adipose tissues. The concentration of the seven higher than average contributing oxylipins in **(A)** murine BAT and WAT (*n* = 7), **(B)** human BAT and WAT (*n* = 8), and **(C)** murine white and brite adipose tissue (*n* = 7). Bars represent mean values and *p*-values are derived from paired **(A,B)** or unpaired **(C)** Wilcoxon test.

The oxylipin profile serves as a potential surrogate measure for the abundance of brown but not brite adipocytes. We asked which metabolites contributed the most to this phenomenon and whether we could identify novel oxylipins associated with the recruitment of brown and brite adipocytes. Therefore, we investigated the contribution of individual oxylipins to the principal components 1 and 2. In the murine adipose tissues, more than two-thirds of the measured fatty acid metabolites contribute higher-than-average to the first two principal components (Figures 3D,F). In contrast, less than half of the compounds did so in the human tissues (Figure 3E). Interestingly, several compounds previously associated with the recruitment of brown and brite adipocytes, namely 9- and 13-HODE (22), the PGI2 degradation product 6k-PGF1α (14, 15), 12-HETE (17) as well as PGE2 (13, 14) contributed higher-than-average in the murine BAT/WAT comparison (Figure 3D). Among those, only 9- and 13-HODE consistently contributed to the discrimination of BAT and brite adipose tissue from WAT (Figures 3D–F), suggesting an association of individual metabolites with the abundance of thermogenic competent adipocytes in a translational context. In line with this notion, the AA-derivatives 11,12-EET and 5,6-EET generated by the CYP pathway, and the LOX pathway products 15-HETE, 5-HETE and its active form 5-oxoETE contributed above-average in all three conditions (Figures 3D–F). However, the abundance of most of these metabolites was exclusively different in murine BAT vs. WAT but not in the other comparisons (Figures 4A–C), confirming the limited discriminative potential of the oxylipin profile in these settings. Only the abundance of 13-HODE in humans and 5-oxoETE in the murine brite vs. white comparison were significantly different in brown and brite adipose tissue compared to WAT, respectively (Figures 4B,C). Interestingly, 11,12-EET, 5,6-EET, and 5-HETE were significantly higher in murine BAT than in WAT (Figure 4A), contradicting the overall trend toward higher total oxylipin abundance in WAT (Figure 2C). This suggests an involvement of these three metabolites in regulation of BAT function. We identified 5,6-EET and 5-HETE as the only two metabolites significantly more abundant in murine BAT compared to WAT that showed at least a similar trend toward a higher abundance in murine brite and human BAT vs. WAT (Figures 4B,C). In line with this regulation, 5-oxoETE, the oxidation product of 5-HETE, also tended to be more abundant in these tissues. Consequently, 5-HETE and 5,6-EET constitute novel oxylipins associated with the abundance of brown and brite adipocytes in a translational context.

## Discussion

Activation of NST in BAT and brite adipose tissue increases energy expenditure and therefore is a potential therapeutic strategy to treat obesity. Within this scope, metabolites of PUFA (especially oxylipins) are discussed as potential effectors. Several studies have associated selected oxylipins with improved BAT functionality (17, 18) or the recruitment of brite adipocytes (13–15) in mice. However, evidence in the human context is scarce. Only the prostaglandin PGI2 has been shown to increase UCP1 expression in cultured murine and human adipocytes (14, 16). Therefore, we screened the abundance of 36 omega-6 or omega-3 PUFA-derived metabolites in human and murine adipose tissues to identify novel compounds associated with abundance of brown and brite adipocytes in a translational context. Based on their respective PUFA metabolite pattern, murine but not human BAT could be distinguished from WAT. Indeed, it has been argued that human supraclavicular BAT resembles murine brite adipose tissue rather than murine BAT, thus comprising a mixture of white and brite adipocytes (7, 23). This is in line with our finding that in mice the oxylipin profiles of brite adipose tissue induced by cold exposure and WAT at thermoneutrality could also not be distinguished. We think of two possible explanations for this observation. First, the oxylipin profile could be a surrogate marker for the thermogenic activity of the respective adipose tissues, since only BAT but not WAT shows increased metabolic activity in cold acclimatized mice (24). However, we did not measure thermogenic activity in our study, and thus lack direct experimental evidence. Second, the oxylipin profile could be a surrogate marker for the abundance of brown or brite adipocytes that markedly increase in abundance upon cold exposure in both BAT and WAT of mice, respectively. However, we speculate that the relative abundance of interspersed brown or brite adipocytes in human BAT and murine brite adipose tissue, respectively, is not sufficient to notably alter the oxylipin metabolite profiles of the whole tissue.

We therefore checked individual oxylipins with discriminative potential between murine BAT and WAT. A set of seven oxylipins with high potential to explain variability between BAT or brite adipose tissue and WAT in mice and humans was identified. Within this set, we could confirm previously reported oxylipins associated with the recruitment of BAT and browning of WAT. As such, LA-derived 9- and 13- HODE were the most abundant oxylipins in both human and murine adipose tissues and had a high discriminative potential. When used at very high concentrations, both compounds sensitize murine white adipocyte progenitors to β_3_-receptor agonist treatment, consequently increasing UCP1 expression in the presence of isoproterenol (22). In our study, the abundance of 9- and 13-HODE in murine adipose tissues gradually decreased with the abundance of brown and brite adipocytes from white to brite to brown. This phenotype may indicate a coordinated regulation of 9- and 13-HODE production to contain thermogenic capacity on reasonable levels upon prolonged β-adrenergic stimulation (1 week at 5°C). However, the sensitizing effect of 9- and 13-HODE was achieved with supraphysiological concentrations of 68 μM (22). Furthermore, the decreasing abundance of 9- and 13-HODE in increasingly thermogenic competent tissues might simply reflect an increased consumption of precursor fatty acids caused by the higher lipolytic and oxidative activity. Consequently, the relevance of 9- and 13-HODE in a physiological context needs further experimental validation. Interestingly, 12-HETE and 14-HDoHE, two LOX products reported to be upregulated in murine interscapular BAT and inguinal WAT upon cold simulation (17), were also high contributors explaining variability between murine BAT and WAT in our study. In contrast to previously published observations, 14-HDoHE was not only lower at 5°C compared to 30°C in BAT but also lacked regulation in WAT (Supplementary Figure 4). Additionally, 12-HETE concentrations were not different between 5 and 30°C in BAT or WAT (Supplementary Figure 4). We cannot exclude an effect of diets differing in the fatty acid composition altering the supply of oxylipin precursor fatty acids. However, we speculate that the lack of regulation of 12-HETE and 14-HDoHE especially in WAT upon cold stimulation indicates that both oxylipins are not implicated in the process of WAT browning *in vivo*.

In line with the scope of our study, we could identify two novel metabolites, which have not been associated with the recruitment of brown and brite adipocytes. Following a translational pattern, the oxylipins 5-HETE and 5,6-EET were more abundant in BAT and brite adipose tissue compared to WAT in mice and humans. 5,6-EET is directly synthesized from AA via the CYP pathway and can activate transient receptor potential vanilloid 4 (TRPV4) channels (25). In contrast, 5-HETE synthesis from AA is a multi-step process involving 5-LOX and glutathione peroxidases (26). Expression of CYP isoforms responsible for 5,6-EET production has been reported for murine adipocytes (27). Further 5-LOX is expressed in human and murine adipose tissue (28–30). Thus, it is possible that both 5-HETE and 5,6-EET are generated endogenously in adipose tissues, although their tissue-specific abundance may be influenced by plasma levels. Considering the different number of brown adipocytes and levels of Ucp1 expression in BAT and WAT, both compounds might be associated with thermogenic capacity. Indeed, both 5-HETE and 5,6-EET are linked to signaling pathways with the potential to regulate the recruitment of thermogenic capacity in adipose tissue. Although 5-HETE is a rather inactive metabolite that and needs further conversion by 5-hydroxyeicosanoid dehydrogenase (5-HEDH) to the active metabolite 5-oxo-ETE, the latter one activates the OXE receptor and PPARγ (31). Activation of PPARγ is one of the strongest inducers of Ucp1 expression (32). Consequently, 5-HETE could by conversion to 5-oxo-ETE activate PPARγ and regulate adipogenesis and Ucp1 expression. Nevertheless, not all PPARγ agonists are able to increase Ucp1 expression in brown or white adipose tissue. As such, the oxylipin and PPARγ agonist 15d-PGJ2 has no effect on the recruitment of Ucp1 in human adipocytes (33). This in in line with our finding that 15d-PGJ2 was virtually undetectable in BAT as well as WAT and did not contribute to the variation in human adipose tissue samples. In contrast to 5-HETE, 5,6-EET could have a negative regulatory effect on the browning of adipose tissues by binding to the TRPV4 channel. This receptor constitutes a negative regulator of thermogenic capacity as mice with a knockout of TRPV4 show increased expression of Ucp1 in WAT in addition to increased total energy expenditure (34). Although 5-HETE and 5,6-EET have the potential to affect browning of adipose tissues, these effects remain to be demonstrated in *in vitro* and *in vivo* studies aiming to clarify the role both oxylipins for the recruitment and function of brown and brite adipocytes.

In summary, we show that bona fide BAT vs. WAT are distinguishable by their global oxylipin profile. Furthermore, we identify 5-HETE and 5,6-EET as novel compounds associated with the recruitment of brown and brite adipocytes in mice and humans. Further studies need to establish whether these oxylipins are mere markers or functional effectors of thermogenic capacity.

## Data Availability Statement

The datasets generated for this study are available on request to the corresponding author.

## Ethics Statement

The studies involving human participants were reviewed and approved by the ethical review board of the Hospital District of Southwest Finland. The patients/participants provided their written informed consent to participate in this study. The animal study was reviewed and approved by the district government of Upper Bavaria (Regierung von Oberbayern) Reference number ROB-55.2-2532.Vet_02-16-166.

## Author Contributions

SD performed the molecular analysis, data analysis, interpreted the results, and drafted the manuscript. SM conceived and designed the study, performed the mouse experiment, contributed to the molecular analysis and data interpretation, and revised the manuscript. MK conceived and designed the study, contributed to data interpretation, and revised the manuscript. TF contributed to data analysis and interpretation and revised the manuscript. E-ZA conceived and designed the study, contributed to data interpretation, and revised the manuscript. CC contributed to data interpretation and revised the manuscript. KV provided human tissue samples and revised the manuscript.

### Conflict of Interest

The authors declare that the research was conducted in the absence of any commercial or financial relationships that could be construed as a potential conflict of interest. The handling Editor declared a past co-authorship with the E-ZA.
