# Supplementary figures and images for: Fatty Acid Metabolite Profiling Reveals Oxylipins as Markers of Brown but Not Brite Adipose Tissue
Source: Front Endocrinol (Lausanne). 2020 Feb 21;11:73. doi: 10.3389/fendo.2020.00073 (PMC7046592; doi:10.3389/fendo.2020.00073)

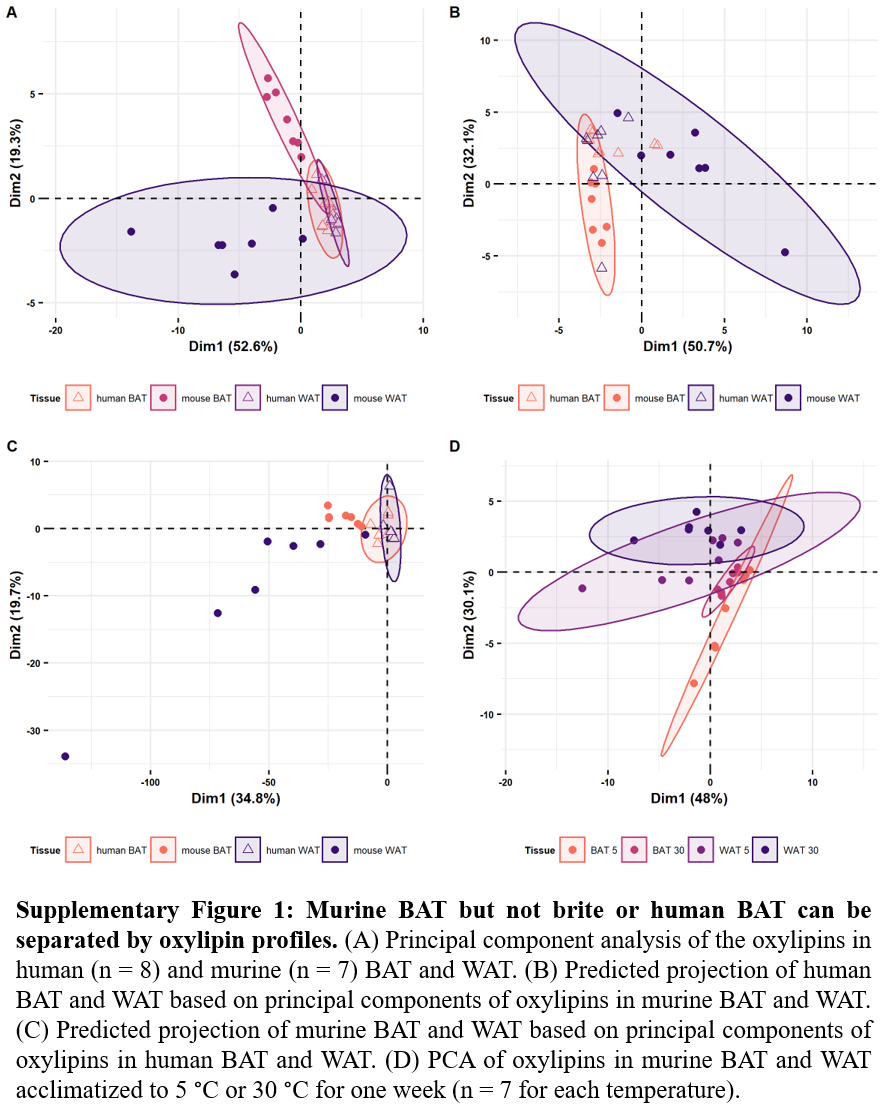

Supplement: Supplementary file 1 [file Image_1.TIF]

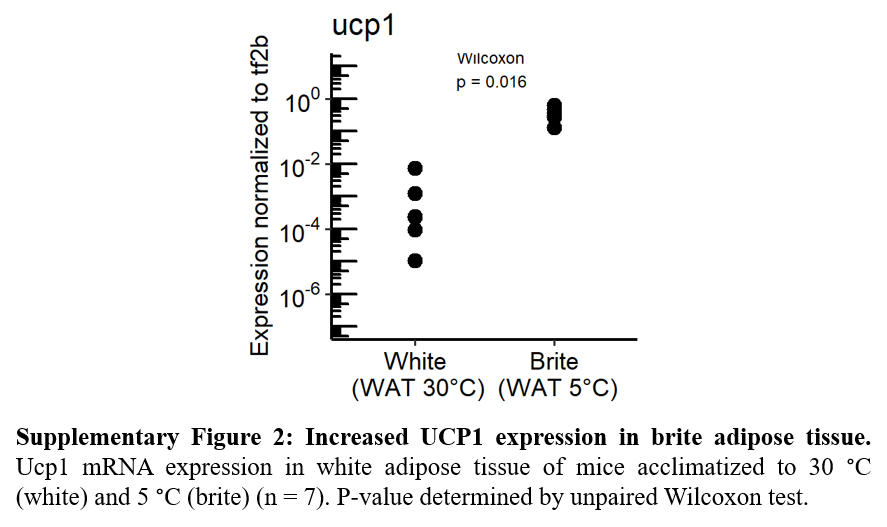

Supplement: Supplementary file 2 [file Image_2.TIF]

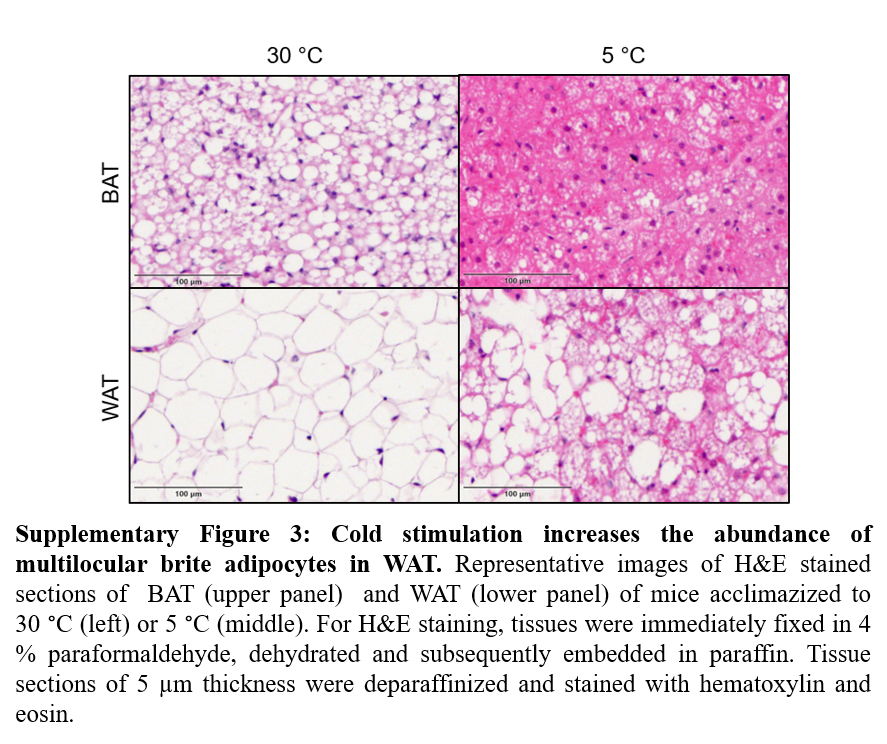

Supplement: Supplementary file 3 [file Image_3.TIF]

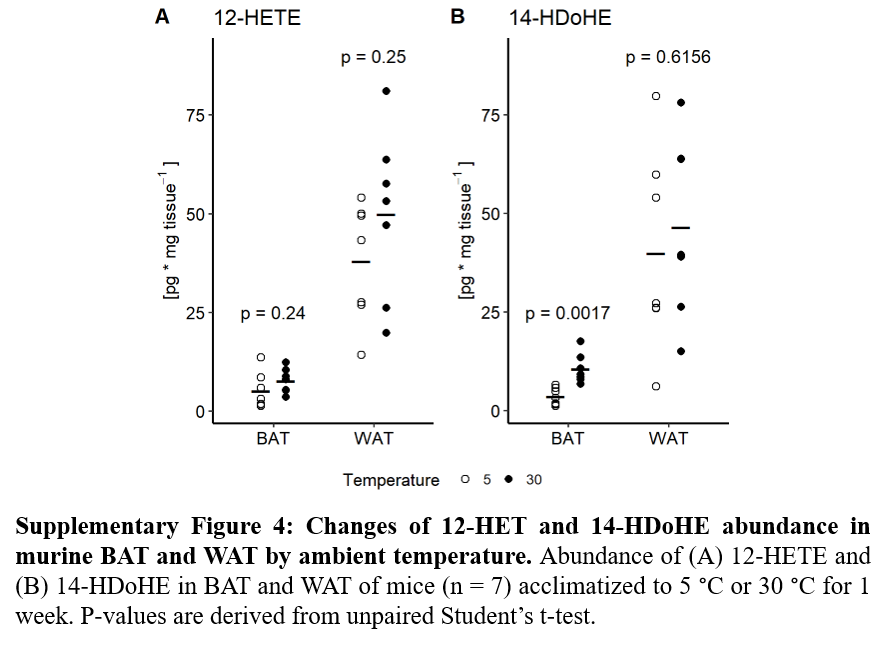

Supplement: Supplementary file 4 [file Image_4.TIF]
